# A Novel Approach for Segment-Length Selection Based on Stationarity to Perform Effective Connectivity Analysis Applied to Resting-State EEG Signals

**DOI:** 10.3390/s22134747

**Published:** 2022-06-23

**Authors:** Leonardo Góngora, Alessia Paglialonga, Alfonso Mastropietro, Giovanna Rizzo, Riccardo Barbieri

**Affiliations:** 1 Department of Electronics, Informatics and Bioengineering, Politecnico di Milano, 20133 Milan, Italy; leonardoandres.gongora@mail.polimi.it; 2 Istituto di Elettronica e di Ingegneria dell’Informazione e delle Telecomunicazioni (IEIIT), Consiglio Nazionale delle Ricerche (CNR), 20133 Milan, Italy; alessia.paglialonga@ieiit.cnr.it; 3 Istituto di Tecnologie Biomediche (ITB), Consiglio Nazionale delle Ricerche (CNR), 20054 Segrate, Italy; alfonso.mastropietro@itb.cnr.it (A.M.); giovanna.rizzo@itb.cnr.it (G.R.)

**Keywords:** EEG, effective connectivity, kurtosis, resting-state connectivity, stationarity

## Abstract

Connectivity among different areas within the brain is a topic that has been notably studied in the last decade. In particular, EEG-derived measures of effective connectivity examine the directionalities and the exerted influences raised from the interactions among neural sources that are masked out on EEG signals. This is usually performed by fitting multivariate autoregressive models that rely on the stationarity that is assumed to be maintained over shorter bits of the signals. However, despite being a central condition, the selection process of a segment length that guarantees stationary conditions has not been systematically addressed within the effective connectivity framework, and thus, plenty of works consider different window sizes and provide a diversity of connectivity results. In this study, a segment-size-selection procedure based on fourth-order statistics is proposed to make an informed decision on the appropriate window size that guarantees stationarity both in temporal and spatial terms. Specifically, kurtosis is estimated as a function of the window size and used to measure stationarity. A search algorithm is implemented to find the segments with similar stationary properties while maximizing the number of channels that exhibit the same properties and grouping them accordingly. This approach is tested on EEG signals recorded from six healthy subjects during resting-state conditions, and the results obtained from the proposed method are compared to those obtained using the classical approach for mapping effective connectivity. The results show that the proposed method highlights the influence that arises in the Default Mode Network circuit by selecting a window of 4 s, which provides, overall, the most uniform stationary properties across channels.

## 1. Introduction

The analysis of the interactions encompassed by different neural sources in the brain, known as connectivity analysis, has become a topic of great relevance in neuroscience. Specifically, the structural, functional, and causal relationships that take place in the brain during neural activity are considered the building blocks to explain how the brain transmits and retrieves neural information [1,2,3]. This plays a major role in understanding neurological disorders, providing an overview of the differences that characterize a pathological condition in comparison to a healthy state [4,5,6,7].

Like many other topics in neuroscience, connectivity analysis has progressed significantly thanks to the advancements in neuroimaging. Brain imaging techniques allow for expressing neural activity in several ways: considering the temporal variation of bioelectric and magnetic potentials and tracking down the flow, or the light absorbance of the blood circulating in the brain [8]. By measuring such quantities, non-invasive data-acquisition methods such as electroencephalography (EEG), magnetoencephalography (MEG) and functional magnetic resonance imaging (fMRI) provide a way to observe the dynamic behavior of the neural activity expressed as multivariate time series from which connectivity among neural sources can be estimated [9].

Setting aside the anatomical connectivity, which looks for structural connections that physically link groups of neurons [10], in some cases requiring invasive techniques to do so [11], we are left with functional (FC) and effective connectivity (EC) as forms of characterizing neural processes from non-invasive measures. The former explores how spatially remote neural populations are being functionally integrated during a brain process, whereas the latter examines the causal relationships and the directed influences exerted among neural sources over the same kind of process. In this context, estimating effective and functional relationships is highly dependent on the temporal reference from which samples are acquired, hence, neuroimaging approaches such as EEG, MEG, and fMRI (to some extent) are appropriate for such a task.

In this way, to estimate the causal influences in multivariate time series (i.e., EC), EEG and MEG provide well-suited data to develop generative models from which inferences of the coupling of different brain regions are made. In summary, functional connectivity shows the distribution of the brain activity assessed by statistically significant values while EC analysis explains the complex elements of the information processing occurring in the brain, which lead to an understanding of how the brain works [3].

Accordingly, the EC studies comprise a wide range of applications such as theoretical constructions for high-resolution EEG recordings [2], the comparison of different effective connectivity measures according to the neural information to be analyzed [12], or the definition of graphical processing approaches for the coupled systems that can be obtained from multivariate time series [13]. Moreover, a range of disorders has been addressed: for example, studies have focused on resting states in long-standing vegetative-state patients [14], on causal relationships among specific areas in the brain associated with the alpha and beta bands during migraine episodes [15], and on patients with treatment-resistant schizophrenia [5], epilepsy episodes in children [16], autism [7] and drug abuse [17]. Other examples of EC analysis include task classification from features extracted from connectivity relationships and applied to the identification of individuals [18], motor imagery prediction [19], object recognition from visual stimulation [20], and the analysis of the brain response to emotional music [21].

EC analysis applied to resting-state conditions has been also analyzed. The work described in [22] provided a detailed investigation of the connectivity exhibited among EEG sources treated in the channel space, where high-density EEG recordings were analyzed in terms of different EC metrics such as Direct Transfer Function (DTF), Transfer Entropy (TE) and Phase Locking Value (PLV). Here, Olejarczyk and colleagues established the significance of the connections by considering weighted adjacency matrices estimated every 20 s to analyze common brain rhythms comprising the alpha, beta, gamma, delta, and theta frequency bands. From the physiological point of view, the authors found that the information flows from the posterior area of the brain towards the frontal area, exhibiting a marked correlation between the central–posterior to the central–frontal region, suggesting the activation of areas involved in the so-called Default Mode Network (DMN) mostly present over the alpha and beta frequency bands.

A similar work that applied graph theoretical analysis to the connectivity of resting-state conditions with open and closed eyes is presented in [23]. Here, the researchers analyzed the alpha, beta, and theta bands by employing the Synchronization Likelihood (SL) to characterize the connectivity using different topological parameters of the network. They analyzed the network according to the SL to understand where the main nodes are located and how they interact by considering the evolution of the resting-state condition every 10 s. They found that by opening the eyes, the connections in the frontal area for the theta band were decreased, similarly to what was observed for the posterior area connecting in a bilateral way to the surrounding zones for the alpha band. This is different from what was found in [22], where a noticeable cluster of sources offered significant connections over these areas in the posterior region for both open-eyes and closed-eyes conditions.

On the other hand, Chen et al. [24] found direct links in the frontoparietal connections characterizing the resting-state conditions with open and closed eyes. While there was observed suppression of the activity over the alpha band with open eyes, they noticed that the connectivity was strengthened in a significant way between the posterior regions in the left hemisphere in comparison to the right one when the signals were analyzed considering segments of 4 s.

Finally, as an example of the analysis of connectivity in age-related brain degeneration, the work described in [25] describes the employment of segments of 2 s and functional connectivity estimators, from which higher connectivity values quantified by the small-world metrics were observed during the open-eyes condition for the alpha band. This also differs from the observations presented previously and highlights the appreciable differences in the results of similar works, which could be linked to the segment duration employed over the methodologies.

All these research works rely on a suitable framework to obtain connectivity measures that explain the causal influences from the neural information. Such a method comprises several steps including preprocessing, where artifacts, noise, and normalization of the signals are performed, and then, the definition of a working domain from the neural sources, which is established either directly from the multivariate time series [15], regions of interest (ROIs) or dipoles [2,26]. From this working domain, the EC calculation is then performed using different metrics such as Granger causality [15], Directed Transfer Function (DTF) [22], Partial Directed Coherence (PDC) [4], and transfer entropy, among others. After that, in some cases, graph-based metrics are employed to characterize the high-degree network generated by the neural sources [23,24], in order to finally perform the statistical analysis used to test the significant connections of the network, providing the final coupled relationships found across the time series as result of the fitting process of a multivariate autoregressive model (MVAR).

Despite this comprehensive methodology, the approaches described in the literature do not provide a framework for the selection of an appropriate segment length to guarantee stationarity to perform effective connectivity analysis. In general, this matter is not usually addressed, and its influence on the MVAR model regardless of its importance has been overlooked. Moreover, the heterogeneity of segment durations employed to estimate connectivity is so diverse that all the works listed so far employ segments that range in the order of milliseconds [12], up to 100 s [7], which could impact the quality of the results obtained, affecting the analysis of connectivity.

For this reason, in this study, we devise a segment-length-selection method that considers the stationary characteristics of EEG signals based on high-order statistical moments and we assess the influence of the segment length on the MVAR model and its corresponding connectivity results, as compared to the conventional approach based on the framework specified above.

In this study, we employed EEG data acquired in the resting-state conditions with open (R1) and closed (R2) eyes to evaluate the implementation of a segment-length-selection algorithm as a preliminary step for effective connectivity analysis. The objective is to select a segment duration that guarantees constant stationary features from the multivariate time series. To do so, an iterative piecewise segmentation of the EEG signals is performed to divide the time series into smaller portions from which kurtosis values are calculated. Then, distributions of the kurtosis variances from the segments are estimated, and a searching strategy is implemented to find the most common segment duration across the EEG signals that maintain the stationary characteristics, not only on each recording but in different neural conditions and subjects.

## 2. Materials and Methods

### 2.1. EEG Dataset

The EEG dataset employed in our study was provided by the Istituto di Technologie Biomediche (ITB) of the Consiglio Nazionale delle Ricerche (CNR). The dataset comprises the EEG time series of 10 healthy subjects. These participants were part of a control group of a clinical research project that evaluated quantitative EEG markers from the brain activity of chronic stroke patients with monolateral upper-limb deficits before and after undergoing a robot-assisted rehabilitation program [27]. The experimental sessions took place at the Presidio di Riabilitazione dell’Ospedale Valduce Villa Beretta, Costa Masnaga (LC), Italy. The project protocol included EEG recordings under resting-state conditions (i.e., relaxation states during open- and closed-eyes conditions) and during motor tasks to characterize and analyze the stroke patients’ evolution during the rehabilitation therapy [28,29]. Written informed consent was obtained from each subject before inclusion in the study. The study was reviewed and approved by the local Ethics Committee at A. Manzoni Hospital, Lecco, and was conducted in compliance with the Declaration of Helsinki.

In this study, two EEG recordings of approximately 5 min (4.77 ± 0.86 min) were analyzed from each subject and comprised the time series of the two resting-state conditions: R1—open-eyes resting state and R2—closed-eyes resting state. This totaled 20 EEG recordings that contained the signals coming from 62 channels that were placed over the scalp using the 10–20 standard system. The Synamps 2/RT system from Compumedics ® Neuroscan™ (Charlotte, NC, USA)was employed for the acquisition and it was configured at a sampling frequency of 1000 Hz with an active power line filtering set at 50 Hz, employing a Notch filter configured at each channel. Out of the 62 channels, the ground electrode CZ was employed to eliminate possible spurious components from the signals, canceling out the noise produced by the ground circuit of the EEG acquisition system.

A preprocessing framework was performed using the EEGLAB toolbox running on MATLAB version 2019a (The Math Works, Inc. MATLAB. Natick, MA, USA) [30], to mitigate noise and artifacts. First, by using EEGLAB’s Artifact Subspace Reconstruction tool [31], the artifacts of the signals were reduced and, in those cases where the affected portions of the signals could not be repaired, such segments were eliminated. Then, a data-cleaning stage was performed by setting up a threshold scheme that considered the density power, signal amplitudes, probability of occurrence, and trend analysis. Finally, Independent Component Analysis and the Multiple Artifact Rejection Algorithm (MARA) were employed to discard portions of the signals that were not compliant with the common features of EEG signals; this was evaluated with a custom neural network embedded in the MARA tool [32]. The independent components that were discarded from the EEG datasets were automatically removed by this tool, following the inherent pretrained parameters of the MARA neural network. In addition, to eliminate noisy portions of the signals, an initial epoching that considered epoch durations of 1 s was employed, and EEGLAB considering the ASR, the thresholds, and MARA removed the portions considered as heavily affected by noise; as a result, the EEG recordings were shortened as shown in Table 1. According to this semi-automatic artifact-rejection framework, bad channels were also discarded following the EEGLAB pipeline as explained in [33].

Table 1 summarizes the main characteristics of the EEG signals’ duration before and after the preprocessing stage described above. As can be noticed, noise and artifacts heavily affected some of the recordings, resulting in a significant reduction in the signals’ duration after the data-cleaning process; in some cases, the proportion of the retained signals was as low as 15%. Hence, in this study, only the clean signals that maintained at least 50% of the original durations were selected to continue the processing. Accordingly, the recordings from subjects 1, 3, 4, and 7 were discarded (highlighted in gray on Table 1), leaving 12 out of 20 recordings from 6 out of 10 subjects available for processing. Table 1 also shows that the number of channels maintained after the artifact rejection was heterogeneous among the recordings and ranged from 55 to 61.

A resampling step was employed after performing the data-cleaning process on the signals to reduce the sampling frequency from 1000 Hz to 250 Hz, which is an acceptable rate considering the frequency information of the alpha band to accomplish the EC analysis [34]. Then, the resampled signals were band-pass filtered using a Finite Impulse Response (FIR) filter that employed a Kaiser window with cutoff frequencies of 0.5 Hz and 50 Hz [35,36]. Finally, the data were common-average referenced. These steps of resampling, filtering, and referencing conclude the digital conditioning stage of the EEG signals. The following sections explain the segment-length analysis and selection based on kurtosis to perform the EC analysis.

### 2.2. Segmentation and Kurtosis Estimation

The proposed segment-length analysis is based on an iterative piecewise subdivision of the signals into segments. From such segments, it is possible to obtain estimations of the dynamical properties of the EEG signals and the nonlinear processes behind them by evaluating the effective connectivity.

**Figure 1:**
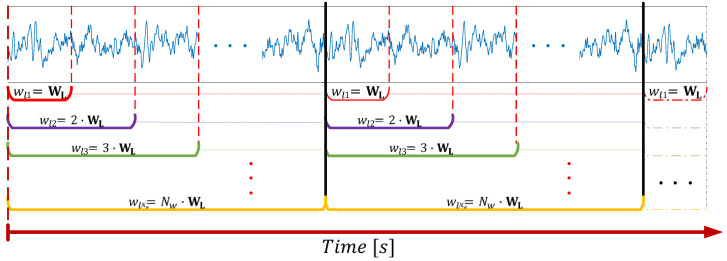
Outline of the segmentation approach performed on EEG signals. (Each color is associated to a specific window length duration: red − *w*_*l*1,_ purple − *w*_*l*2,_ green − *w*_*l*3_, and so on).

The segmentation approach is summarized in Figure 1_._ Let W_L_ be the basis window length defined as an elemental duration of the segments in seconds, and *N*_w_ be the total number of windows (equivalent to the number of segmentation operations) considered to perform the iterative segmentation. Then, according to these parameters, *h* is defined as the longest segment duration following that *h* = *N*_w_∙W_L_, thus holding that 0 < W_L_ ≤ *h*. By considering the total duration of the recording (***t***), as well as the variable *w*_*l*i_, used to keep the value of the segment duration for a specific segmenting step (*i* = 1, …, *N*_w_), at each iteration, a matrix of size *t*/*w*_*l*i_ by *w*_*l*i_ ∙ *fs* is built and contains the segmented signal with non-overlapping segments. The variable *w*_*li*_ refers to the segment duration according to the segmenting step iteration, so that *w*_*li*_ = *i* ∙ W_L_, ∀*i* = {1, …, *N*_*w*_}, and *fs* corresponds to the sample frequency (i.e., 250 Hz in this case).

In summary, the iterative process for the segmentation of a signal is explained by its sequential splitting according to the window length (*w*_*li*_) whose duration is increased at each iteration by a factor defined as a multiple of the basis window (W_L_) given *i*. In this way, the signal of duration ***t*** is divided into non-overlapping pieces, each one of length *w*_*li*_. The resulted segments are then stored in matrix form and are organized in chronological order. The procedure is repeated for the original signal *N*_*w*_ times, producing a total of *N*_*w*_ matrices of segments for each of the signals that compose the dataset. Since the number of windows is directly related to the window length, *N*_*w*_ must be chosen according to the physiological characteristics of the brain activity under analysis and the frequency information that we want to cover with the selected windows. However, this process can be trivial if *N*_*w*_ is set large enough so that the different windows comprise the needed frequency components to be analyzed.

The sequential process is performed until *i* = *N*_*w*_, whose value is defined beforehand. From this approach, it can be easily noted that each segment is composed of a sequence of samples generically defined by the vector $w_{li,j}=\left(x_{j},\;x_{j+1},\;x_{j+2},\ldots ,x_{j+i\cdot {\mathrm{SW}}_{L}-1},\;x_{j+i\cdot SW_{L}}\right)$, where x_k_ (for k=j,  j+1, … j+i SWL) generically refers to the components of the EEG segment *w*_*li*__,__*j*_. Here *j* corresponds to the index of the sample where the segment starts with respect to its occurrence in time, and SW_L_ is the number of samples contained in the basis window W_L_ (i.e., SW_L_ = W_L_∙ *fs*). Thus, *w*_*li*__,_ defines the data vector resulting from a specific segment, a data block formed by a number equal to *i* ∙ SW_L_ samples that initiates at the time instant corresponding to the index *j*.

The segments that belong to a row of non-overlapping windows (shown in the lower part of Figure 1, represented by the horizontal brackets) form a new matrix containing the windowed signal according to *w*_*li*_. Each row of this matrix encloses a single segment from which different statistical measures such as the mean, variance, skewness, or kurtosis can be estimated. From these statistical moments, it is possible to evaluate the stationary characteristics of a signal as a function of time given the time interval definitions considered in the segmentation approach.

From the previous characterization, each matrix containing the signal’s segments associated with a channel (*chn*) that belongs to the EEG dataset follows the definition shown in Equation (1).

(1) $$W_{matrix}\in {\mathbb{R}}^{t/w_{li}\;x\;w_{li}\cdot f_{s}}\to \;s.c\in {\mathbb{R}}^{t/w_{li}}\;\;,\forall chn=\left\{1,\ldots ,M\right\}$$

where *W*_*matrix*_ is a matrix containing the segments of a signal and 𝑠.*c* stands for the statistical characteristic whose values are being mapped into. From Equation (1), it is noted that the statistical characteristic space has a dimension of *t*/*w*_*li*_, corresponding to a vector that represents the calculated statistical moment and whose components are each related to a segment at a specific time interval, i.e., each vector component is attributed to a time interval represented by a segment. In this way, it is possible to account for the variation over time of these statistical characteristics considering different window ranges and the given matrices.

For our specific case, we rely on the kurtosis (Equation (2)) to account for the stationarity of the segments. This equation calculates the fourth-order central moment by estimating the expected value of the fourth power of the difference between the time series (x) and its mean ($\mu_{x}$), and applying the normalization by dividing it by the squared variance of x and subtracting the kurtosis value of a pure Gaussian distribution (i.e., 3), so that the offset, known as kurtosis excess, accounts for the difference between the kurtosis of the time-series segment (x) and a strict stationary series that follows Gaussian distribution.

(2) $${K^{\prime }}_{x}=\frac{E\left[{\left(x-\mu_{x}\right)}^{4}\right]}{\sigma_{x}^{4}}-3$$

High-order moments such as kurtosis contribute to the process characterization. Unlike the first- and second-order statistical moments that are limited (in our case) by the zero-mean characteristic of the time series, kurtosis accounts for the existing difference of a normal distribution when it is compared to the Probability Density Function (PDF) formed from the samples that belong to a segment. This fourth-order moment can be employed to determine the non-stationarity behavior exhibited by a segment of fixed duration [37]. Since random processes are assumed to be stationary, and their distributions follow a Gaussian density, then, by evaluating how different segments’ PDFs differ from the normal distribution, the non-stationarity proportion of the segment can be estimated.

Under these assumptions, Equation (1) can be rewritten as:

(3) $$W_{i,chn}\in {\mathbb{R}}^{t/w_{li}\;x\;w_{li}\cdot f_{s}}\to K_{i,chn}\in {\mathbb{R}}^{t/w_{li}}\;\;,\forall chn=\left\{1,\ldots ,M\right\}$$

where *K*_*i*__,__*c*__*h*__*n*_ is a vector that contains the kurtosis excess estimated at each segment from the *W*_*i*,c__*h*__*n*_ = *W*_*matrix*_ at iteration *i* and channel *chn* (i.e., a vector of kurtosis whose components are calculated from each row of the windowed matrix). Then, each component of the vector *K*_*i*__,*h*__*n*_ explains the degree of non-stationarity of a segment at a specific time interval bounded by the duration *w*_*li*_ on each channel.

### 2.3. Kurtosis as a Feature

The fourth-order central moment characterizes each segment of the time series in our approach; this is a feature derived from shorter portions of the data and explains the dynamic change of the stationarity from segment to segment. Therefore, different segmentation conditions provide different amounts of information about non-stationary characteristics requiring comparing kurtosis values from a single channel, a complete dataset, and finally, among conditions and subjects.

**Figure 2:**
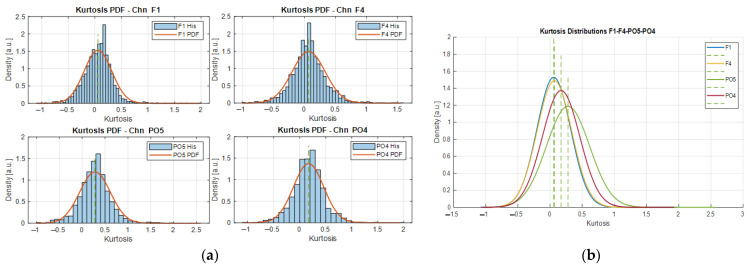
(**a**) Individual kurtosis distributions for the channels F1, F4, PO5, and PO4. (**b**) Superimposition of the kurtosis distributions. Estimated for subject 10 during closed-eyes resting-state condition.

From Equations (1) and (3), it can be noticed that the resulting kurtosis vectors have dimensions varying according to *t*/*w*_*li*_; in consequence, an interpolation step is performed to guarantee the same size across the vectors from which the kurtosis PDF is estimated and comprises the kurtosis values of a channel subjected to the *N*_*w*_ segmenting iterations. Figure 2 shows the kurtosis distributions of 4 different channels. As can be observed from Figure 2a, the kurtosis distributions of different channels follow a Gaussian-like density as depicted by the PDFs estimated from the segmentation process of the signals associated with the channels F1, F4, PO5, and PO4. The kurtosis distributions in Figure 2a are the result of the iterative segmentation process of the signals considering a basis window length WL = 1 s, for 1 ≤ *i* ≤ 10, yielding 10 kurtosis vectors as result, from which the PDFs are fitted. The expected values on each distribution correspond to the most likely kurtosis expressed by the signal over different segment lengths and they are used to assess the stationarity of the signal as a function of the window length. The examples in Figure 2a are related to some representative EEG sources, i.e., the frontal (F1, F4) and posterior (PO4, PO5) electrode locations.

As can be noted from the densities in Figure 2a, they have similar shapes and their distributions span comparable ranges (i.e., approximately −1.5 ≤ K ≤ 2, with expected values in the range of 0 to 0.5, as shown by the green dashed lines in Figure 2b. Where K corresponds to the kurtosis value). In this specific example, the relationship that exists between the frontal channels F1 and F4 is evident as the PDFs are nearly the same. This is an expected behavior since the electrodes’ locations on the F1–F4 channels are close to each other relative to their positions from PO5 and PO4.

Furthermore, considering that the alpha rhythm (comprising frequencies from 8 to 13 Hz) is more noticeable over the occipital area during resting-state conditions, then the EEG data acquired by the channels PO5 and PO4 should evidence an appreciable increment of the power spectrum under this frequency range. Moreover, since the frequency patterns are not uniformly distributed over the scalp, it is expected to find differences of such magnitudes over the same area; in addition, those differences could influence the non-stationarity behavior of the signals, which could be the reason for the slight variations of the kurtosis PDFs in Figure 2b. As explained in [23], the posterior region (covered by the occipital area where PO5 and PO4 are located) has an emergent pattern of connectivity directed to the frontal–parietal regions, suggesting as well non-uniform stationary behavior in this area that may be explained by the kurtosis distributions of these channels.

Moreover, by superimposing the kurtosis distributions from different channels, we can observe how distant their expected values are with respect to each other, from the point of view of each PDF (as shown in Figure 2b). If more distributions are compared, then we can find subsets of channels whose expected kurtosis values are closer than others, hence there exists a probability range that gathers most of these expected values associated with a specific segment duration. With this information, we design a searching strategy to find common segment durations across channels that exhibit similar stationary characteristics.

### 2.4. Kurtosis Variance and Searching Strategy

The proposed strategy for defining the segment length is based on the search for kurtosis values that are likely to be found across the PDFs estimated from the multivariate time series of the different EEG signals. Specifically, from the time series, different segment lengths are evaluated to find a window duration, common across channels, that guarantees similar stationary characteristics to perform effective connectivity analysis. From the distributions of the kurtosis of different channels (as shown in Figure 2), their corresponding variances are computed as a function of the segment length, i.e., $\sigma^{2}\left(K_{i,chn}\right)$. The main advantages of using kurtosis variance are:

1. The kurtosis vector (*K*_*i*_) generated for each channel is re-expressed as a single value representing the squared deviation from the expected mean magnitude considering a specific segment length. In further processing, this is computationally less expensive than a vector of *t*/*w*_*li*_ components. The data re-expression can be explained as follows:
  (4) $$K_{i,chn}\in {\mathbb{R}}^{t/w_{l}}\;\;\to \;\sigma^{2}\left(K_{i,chn}\right)=K_{\sigma_{i,chn}^{2}}\in {\mathbb{R}}^{1},\;\;\;\forall Chn=\left\{1,\ldots ,M\right\}$$
2. Similar means and variance values from different segment lengths enable a comparison of the dispersion observed on a dataset containing different signals, as exemplified in Figure 2b.

**Figure 3:**
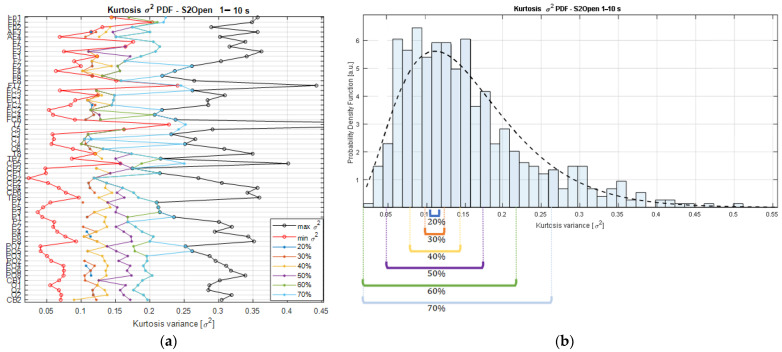
Searching space boundaries relative to the kurtosis limits, estimated for subject 2 (S2) in open-eyes condition. (**a**) At the channel level. (**b**) From the distribution point of view.

The searching strategy consists of finding the smallest range of kurtosis variance that contains the expected values of the kurtosis PDFs (i.e., the mean) estimated across channels in each recording. As an example, Figure 3b shows the kurtosis variance distribution of the $K_{i,chn}$ vectors from one of the participants of the study. The PDF from the kurtosis variance values is fitted by a Chi-square (*X*2) distribution (black dashed line); therefore, the resulting density is estimated from the *Kσ*^2^ matrix that holds the kurtosis variances with respect to the multiples of the basis segment length (*w*_*li*_, for *i* = {1,…,10}) for each channel of the EEG recording. This means that the matrix *Kσ*^2^ is a result of the concatenation of the $K_{\sigma_{i,chn}^{2}}$ vectors. This means that *Kσ*^2^ contains the variances estimated from the kurtosis values of each channel’s time series segmented at various scales, from 1 s to 10 s, producing a matrix of a maximum size of 62×10 components, considering that our dataset comprised a total of 62 EEG signals, but that was reduced in some datasets after performing the signal preprocessing. In this way, the variance searching algorithm is defined as Algorithm 1:
**Algorithm 1:** Variance searching algorithm**Input:***Kσ*^2^1.$pdf\;\leftarrow \;MLE\left(K\sigma 2,\;\chi 2\right)$2.$pk\;\leftarrow \;max\left(pdf\right)$3.$L_{b}\leftarrow \;pk,\;H_{b}\;\leftarrow \;pk$
Loop4.
 
$L_{b}\;\leftarrow \;L_{b}\;–\;c$


 
$H_{b}\;\leftarrow \;H_{b}+c$
5.
 
$S_{K\sigma^{2}}=find\left(K\sigma^{2}\right)\;such\;that:\;L_{b}\;\leq \;K\sigma^{2}\leq H_{b}$
6.
 
$VSelChn\;\leftarrow S_{K\sigma^{2}}\;\left[\min\left(t_{w,chn}\right)\right]$


 
$IF\;count\left(VSelChn\right)>threshold1.$
7.

  
$Avar\;\leftarrow \;mean\left(VselChn\right)$




   
IF Avar ≤ threshold2.
9.



    
Append Avar to SELECTION

Until k iterations are reached

Algorithm 1 shows how to perform the variance range searching strategy. It receives the *Kσ*^2^ matrix as the input, from which the PDF is estimated considering the Maximum Likelihood Estimation (MLE) method by fitting a Gamma distribution. After the peak value of the distribution (pk) is found, it is used to initialize the lower and higher bounds of the variance range searching interval ($L_{b}$ and $H_{b}$). Then, a constant value c, that sets the searching rate, is subtracted and then added to the lower and higher boundaries, respectively, so that a searching interval is initialized. Now, the kurtosis variance values from the *Kσ*^2^ matrix that are under the searching interval $\left(L_{b}\;\leq \;K\sigma^{2}\leq H_{b}\right)$ and that correspond to the shortest segment duration on each of the channels are selected at the actual iteration and stored in the variable (VSelChn). Since the rationale is to maximize the number of channels that exhibit the same stationary characteristics, first, the number of channels that have at least one segment within the kurtosis variance searching limits is computed and, if the count is less than 50% (threshold 1) of the total number of channels in the dataset, then the algorithm restarts the searching by increasing the interval limits and setting it up as the new searching range. Then, the average of the variances is calculated, and if its value is lower than a second threshold (set at 40% of the maximum variance per channel registered in the matrix), then it is guaranteed that the selected channels exhibit stationary characteristics bounded by this threshold, assuring only minimum variations of the stationary features across the selected channels, thus allowing to find common features over the channels. The channels that meet these bounds are stored in the SELECTION variable, and their durations are sorted out with respect to their variance values.

### 2.5. Searching Domain

Starting with the *Kσ*^2^ matrix, it is possible to rank the variances from the lowest to the highest on each of the channels composing a dataset, and the min and max values per channel are computed to establish the absolute kurtosis variance limits relative to a segment duration. In the same way, the intermediate values are found, and their corresponding durations are associated with a specific proportion of the variance limits, as shown in Figure 3a.

As can be observed from Figure 3a, the lower the percentage threshold, the closer the selected *Kσ*^2^ values are to the min bound and, consequently, the lower the number of channels that will meet the kurtosis variance requirements. A trade-off is necessary as higher percentage threshold values will lead to a higher number of channels but, at the same time, a higher dispersion among the channels, as shown in Figure 3b.

In this sense, sorting the segment durations that are masked out by the percentage thresholds allows us to identify common durations covered by the area of the PDF bounded by the interval limits. As result, we obtain a series of segments that are ordered by both the duration and the relative variance magnitude that they exhibit; this information is used to categorize them accordingly. Then, counting the segment durations that are found at a specific percentage threshold quantifies the number of channels that share similar stationary properties according to their kurtosis variance magnitudes, and, if a cutoff value is established to set a limit on the minimum number of channels expressing the *Kσ*^2^ values within a percentage range, then the searching strategy returns the segment durations meeting these requirements. In this way, the searching strategy shows the segment durations that, at a specific percentage threshold, gather the required number of channels exhibiting similar stationary characteristics. In these terms, the trade-off is solved by performing the selection of the segment length with the lowest percentage threshold reaching the minimum requirement for the number of channels.

To this extent, by considering different percentage thresholds and the minimum number of channels within those limits, it is possible to graphically check the areas covered by the searching interval, and how the selection of the segment duration is derived from the statistical characteristics coming from the dataset, specifically from the kurtosis of the segments. 

### 2.6. Directed Transfer Function

To develop the generative model necessary to explain the directed influences and the relationships that exist between different neural regions, several measures that quantify the existing coupling among sources considering the temporal information from EEG recordings can be employed [1]. Measures based on time series relying on Granger causality and its variants in the frequency domain are the most common choices in effective connectivity analysis for EEG data [12]. In this approach, we employed the Directed Transfer Function (DTF) since it is a measure proven to be more appropriate to be applied to signals registered on the scalp as demonstrated by Ku and colleagues [38]. The quantification of the DTF is defined as shown in Equation (5).

(5) $$DTF_{ij}=\frac{H_{ij}\left(f\right)}{\sqrt{\sum_{m=1}^{n}{\left|H_{im}\left(f\right)\right|}^{2}}}$$

where the matrix H contains the spectral and phase information of the sources i,j, from which causality is assessed. The DTF value is a complex measure and provides a metric of the total information that has been flown from the source j to i, being normalized by the total inflow received by i. In this sense, the DTF detects the direct influence of one or several signal sources in the channel of destination [34].

**Figure 4:**
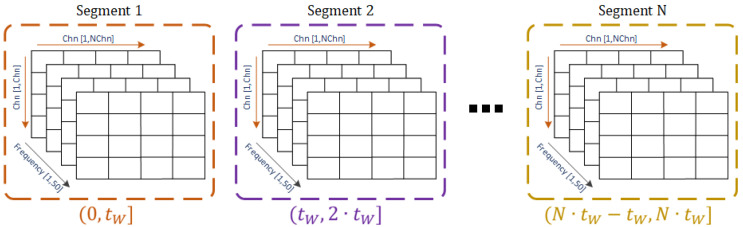
3D matrix of the segments obtained from a generic selected window ($t_{W},\ldots ,\;N\cdot t_{W}$) containing the DTF connectivity values among channels (Chn) at each frequency.

If we consider distinct sources and destinations, defined from the EEG channels, then, Equation (5) is the starting point from which the EC calculation is repeated pairwise on the signals composing the EEG recording. In this way, a relationship among the channels is produced and displayed in matrix form, where each row–column component corresponds to the DTF value estimated on the source and destination signals (j to i), respectively. Since the EEG data are time-dependent, then each of the DTF matrices should refer to a specific time interval to characterize the flow of information at each frequency and comprises components from 1 Hz to *fs*/2. Hence, in this sense, the selected window length derived from our approach is employed to calculate the connectivity matrices along with the signals at every time interval. Figure 4 shows the resulting 3D matrix that comprises the EC values among channels calculated at each frequency on the EEG blocks after segmenting the signals with the selected window duration. The value $t_{W}$ refers to the duration of the selected window, and the segments indicate the time interval along with the EEG signal product of the segmentation considering a generic selected window.

Finally, to assess the significance of the connectivity among channels, the t-test is applied to the DTF blocks that characterize a desired frequency range (performed by averaging elementwise the EC values of the DTF matrices over a desired frequency range, e.g., alpha band), then, only the connections that have a *p*-value less than 0.05 are considered significant and their connectivity relationships are maintained for further analysis. Finally, considering the complexity of the DTF matrices, the statistically significant relationships gathered in these matrices are treated as adjacency matrices, from which graph theory indices are calculated to characterize the network of effective connections.

### 2.7. Network Measures: Graph Theory Indices Applied to EEG Data

The use of the adjacency matrices to encode the significant connections as a result of the DTF estimation across the channels provides the raw structure that shows the effective connections on the channels. This can be difficult to interpret due to the high density of connections that are considered significant from the adjacency matrix. In this way, it is useful to consider graph theory measures to perform a characterization of the connectivity in the network that is able to show hidden structures, and central nodes that participate more in the transmission of information and clusters that could characterize the brain activity that is being investigated, i.e., the resting-state conditions with eyes open (R1) and eyes closed (R2).

As explained in [39], 4 broad classes of graph measures can be distinguished: the basic measures that reflect the importance of a node (channel) in the network by considering the number of connections it has with other nodes (i.e., the degree), the graph density that measures the actual number of connections in the network and that can be expressed as the percentage of links present in the network being 0% when no connections are considered in the graph and 100% when all the significant links are shown, and finally, the strength, which accounts for the amplitude of the connection between two nodes, e.g., the DTF magnitude registered for the pair channel i,j in the matrix.

The second class of measures is the so-called measures of integration, which account for how effortless the communication between the channels is performed. In this category, there are different measures that help to estimate this. The shortest path length between two channels, as its name indicates, calculates the line with the minimum length that connects two nodes on a surface, in this case given the topographic characteristics and the placement of the electrodes over the scalp of a person. Its value is defined for every pair of nodes, and given the high density of nodes that form a network of electrodes, the average shortest path length is used to characterize the typical separation between the nodes.

Conversely, the global efficiency accounts for the inverse of the average shortest path length and indicates the capacity of a network to support the information flow, and in the case where networks are not fully connected, it provides a better representation of the integrative communication characteristics among the nodes since, unlike the average shortest path length, the global efficiency does not diverge to infinity when a connection is not present in the network. This provides a useful way to account for how easy the communication among the present nodes is, since the adjacency matrices in our case are not fully connected, considering that they only contain the statistically significant connections.

The third category of graph parameters is the so-called measures of segregation that characterize the independence of local structures found within the network, given the formation of groups that are interconnected, i.e., clusters of nodes. The clustering coefficient accounts for the channels connected to a node that are interconnected to each other. Another measure of segregation is the local efficiency, which is defined as the efficiency among the neighbors of a node.

Lastly, the importance of a node in the network is estimated by considering the betweenness centrality, a parameter that quantifies how central is a node in the information flow considering the integration and effective connections produced within the structure. This measure calculates the number of the local short paths connected to a node and that represent the importance of a channel in the network.

The density parameter is a basic measure that quantifies the fraction of actual connections that are present on a network. When an adjacency matrix is calculated, it summarizes the effective directed connections among the channels that are significant in statistical terms, as explained above. Then, it is most probable that its density is less than the maximum number of possible connections on the network, defined as $N\;\left(N-1\right)$, N being the total number of channels (i.e., 62). Thus, the density of a non-fully connected network, given the statistically significant relationships condensed in the adjacency matrix, will never be equal to $N\;\left(N-1\right)$.

In these terms, the density is constrained by the number of significant connections of the adjacency matrix whose elements’ magnitudes can be sorted from lowest to highest in order to generate the “cost” variable, which is used as the independent variable from which the remaining graph-based parameters are calculated, and by these considerations, they are defined as a function of the number of actual connections in the network. By sorting out the magnitudes, the cost represented as the proportion of connections encodes a linear scale from the highest to the lowest magnitudes. In this way, 1% of the cost comprises the number of connections in the network that have a magnitude larger or equal to the 99% of the maximum DTF value found in the adjacency matrix. The same applies to the other percentages up to reaching 100%, whose cost comprises all the significant connections regardless of the DTF magnitudes on the matrix.

## 3. Results

### 3.1. Selection of the Window Duration

**Figure 5:**
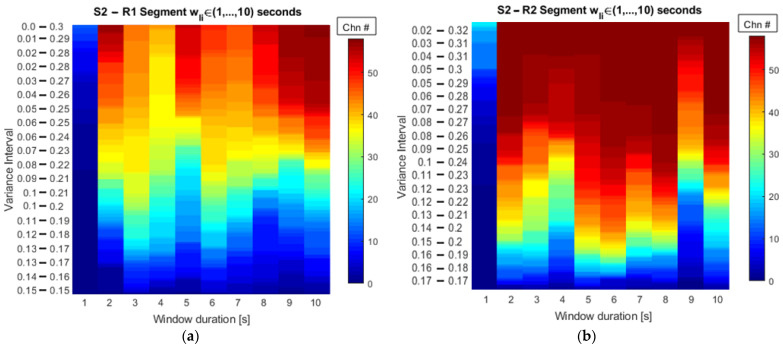
The number of channels as a function of the segment length and the searching interval in terms of the kurtosis variance. (**a**) S2 Open-eyes resting state. (**b**) S2 Closed-eyes resting state.

By applying the searching strategy and continuing with the example depicted in Figure 3, the number of channels that meet the kurtosis variance criteria as a function of the corresponding searching interval and the window length can be computed for the two resting-state conditions. The results are shown in Figure 5.

Figure 5 shows that, as discussed previously, the number of selected channels increases if the variance interval is enlarged, reaching the total number of channels at wider variance intervals. Table 2 reports the exact number of channels from a recording that share similar stationary characteristics as a function of the window duration, for open (R1)- and closed (R2)-eyes resting states for the same subject (S2) following what is shown in Figure 5. The numbers in red in Table 2 (51 and 39) correspond to the number of channels with a similar stationary value associated with the selected segment duration for this EEG recording; according to the threshold for the minimum number of channels set at 75% and 65% of the total number of time series of R1 and R2, respectively.

As can be observed in Table 2, for the open-eyes state (R1 columns), the number of channels at a percentage level of 40% is significantly inferior compared to wider searching intervals. By considering the limits on the kurtosis density from Figure 3b, the 40% and 50% ranges cover the most probable kurtosis variances of the whole dataset. Hence in Table 2**,** at 40% with a window length of 3 s, only 24 channels share the kurtosis variance from that range. Following the same logic at 50% of the variance, the number of channels increases to 34, corresponding to ~58% of the total of channels from the dataset. Moreover, by evaluating a segment duration of 5 s, it is observed that 15 and 20 channels are found in the variance intervals of 40% and 50%, respectively. In this way, looking at the *Kσ*2 PDF using a segment of 5 s allows the selection of 51 channels (~80% of total channels) that share a variance in the range of $0.028\;\leq \;K\sigma^{2}\;\leq \;0.272$. This comprises a probable proportion of the density.

Looking at the data of S2 during the closed-eyes resting-state condition (Table 2 R2 columns), the kurtosis variance proportions for the EEG dataset that was used here are also shown. Following the same analysis, in this case, kurtosis variance intervals comprising relative proportions lower than 50% did not group as many channels as in larger proportions. The windows of 2, 5, and 6 s for the 50% interval ($0.131\;\leq \;K\sigma^{2}\;\;\leq \;0.211$) grouped 65%, 73.3% and 80% of the total of the channels that composed the recording, which suggests that any of these window durations maximize the number of signals with similar stationary characteristics. However, considering the assumptions explained in the methodology, it is required to have a segment length as short as possible that groups many or all the channels. Thus, in this case, a window of 2 s is the one selected for this subject in the closed-eyes resting-state condition. As a reference, the red dashed line in Figure 5 depicts the 50% threshold and graphically shows the number of channels gathered at each window duration for both resting states.

Table 3 summarizes the results obtained for the six subjects. For each of the subjects at each resting-state condition, the table shows the kurtosis variance range limits that provided a sufficiently high number of channels (according to the threshold for the minimum number of channels) with similar stationary characteristics and the resulting window length. The variance percentage indicates what proportion of the maximum kurtosis variance is featured by the selected window duration.

By replicating the same analysis for the remaining 11 recordings composing the overall dataset, the results in Table 3 are obtained. From this table, it is noticed that a window of 4 s is the most common segment length across the EEG data coming from the six subjects. From these results, the kurtosis variance percentage on average corresponds to 40% of the total, providing a significant reduction in the spread of the kurtosis values among the EEG signals from the recording. This, in addition to the 69% of channels that exhibit similar stationary characteristics, reaches the objective of maximizing the number of channels while minimizing the variability of the stationary measures quantified by the kurtosis. This is achieved by analyzing the recordings in relation to the characteristics of each specific dataset.

### 3.2. Effective Connectivity

**Figure 6:**
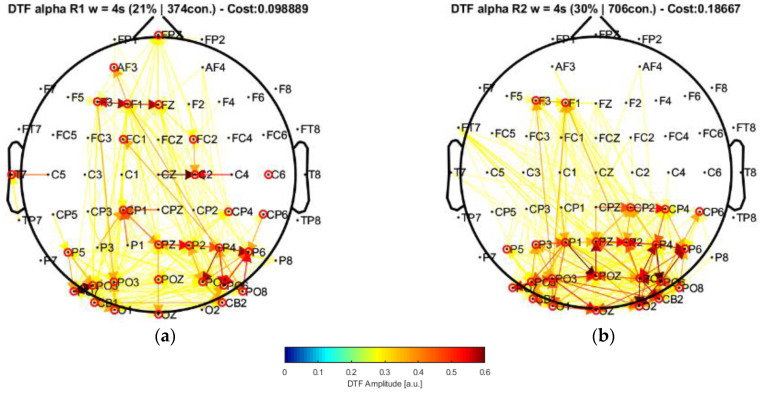
Effective connectivity diagrams considering a window of 4 s (**a**) for the eyes-open state and (**b**) for the eyes-closed state estimated for the 8–13 Hz frequency band (Alpha rhythm).

Figure 6 shows the results of the connectivity for both resting-state conditions when a window of 4 s is applied and analyzed for the alpha frequency band (8–13 Hz).

Figure 6 depicts the EC that characterizes each of the resting-state conditions from the 12 recordings considered for our approach. The strength of the connections is color-coded, and the arrows highlight the directionalities and the relationships among the EEG channel sources. Yellow colors transitioning to orange, red, and finally brown/black, show the connectivity strength in the network from low to high according to the magnitudes of the Directed Transfer Function (DTF) [40,41]. Figure 6 shows the results by establishing the statistically significant connections among the channels that monitored the EEG potentials generated over the scalp when subjects were in a resting state (see Section 2.6).

According to the results from the window-selection approach (see Table 3), a window of 4 s was employed to quantify the connectivity and characterize the influence of different neural sources over the scalp since it was the most common segment duration across subjects and conditions. The directional influence of the connectivity was quantified according to different network measures as shown in [39]. From these network measures, basic, integration, segregation, and centrality quantities were calculated from the adjacency matrices considering the DTF estimations from the signals as explained in Section 2.7. The DTF values were estimated considering the window of 4 s using the Source Information Flow Toolbox for EEGLAB in MATLAB [42], and then, the most significant neural sources represented by the EEG channels were found by integrating the network measures and establishing the central nodes according to the neural process under analysis. Such a procedure was carried out considering the methodologies presented in [14,22,23,24,43].

**Figure 7:**
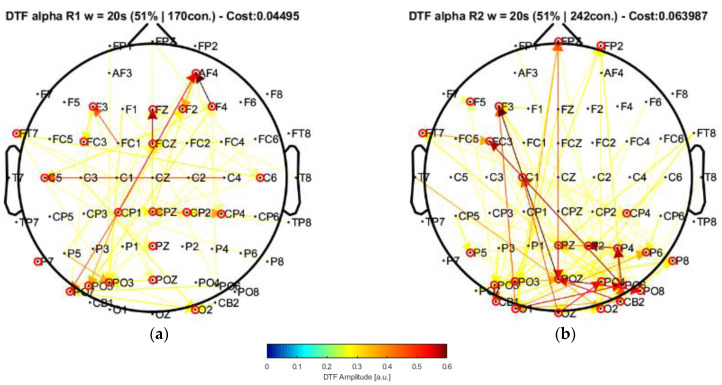
Effective connectivity diagrams considering a window of 20 s. (**a**) for the eyes-open state. (**b**) for the eyes-closed state estimated for the 8–13 Hz frequency band (Alpha rhythm).

Conversely, let us now consider the EC patterns by employing a 20 s window according to the methodology explained by Olejarczyk and colleagues [22]. In this regard, Figure 7 shows the connectivity diagrams obtained from such segment length.

The connectivity diagrams in Figure 6 and Figure 7 show the significant effective connections derived from the adjacency matrices formed by the statistically significant relationships at a cost of 21%, 30%, and 51% of the maximum value of the DTF matrix (for R1 and R2 from the windows of 4 s and 20 s, respectively), which means that only the connections that exhibited a DTF magnitude higher or equal these values with respect to the highest DTF element (~0.6 in all conditions) are being plotted in the graph. The intersection of the selected channels from the graph measures according to [14,22,23,24,43] provides a list of nodes that can be considered as central elements that actively participate in the network. These selected nodes are highlighted by the red circles around their topographic locations in the graphs. Moreover, the directionalities are also depicted in the graph and show how the information is being directed to specific areas from different channels given some identifiable clusters of channels observed in the connectivity diagrams.

From the graphs in Figure 6, it is possible to note that there are clusters formed by some of the electrodes that exhibit a significant increment of their connections. This is the case of the closed-eyes condition in Figure 6b. The electrodes located at the posterior part of the scalp formed by the occipital (Ox), parietal (Px), and central–parietal (CPx) electrodes appear to be more involved in the connectivity process. In this region, the internal connections are very evident in terms of strength and number of connections. In addition, the salient connections with other areas such as the frontal region are shown as well. These connectivity diagrams for the closed-eyes condition provide some insights into the directionality of the connections. In this way, besides the evident internal network occurring in the posterior area of the brain during the eyes-closed condition, an influence is developed from this region towards the frontal area. In the case of the eyes-open condition, the pattern of connections is less consistent, in other words, they are not as structured as in the eyes-closed condition and the strengths from the central channels that participated more in the network were different as well.

For comparison, Figure 7 shows the connectivity diagrams by employing a window of 20 s. As can be observed, the central channels can be grouped to form clusters, which are used to identify the changes in the flow of information not only in the local level given the topographic location of individual electrodes but in a more general view considering complete regions that highlight the active areas in which the connectivity is being produced inside the group of nodes and between these areas. Considering the node grouping, there is observed a set of channels that participate in the EC. Specifically, the channels grouped for the closed-eyes condition (Figure 7b) show a significant influence originating in the posterior region of the brain from which most of the connections are generated. The channels located in this area present local connections and provide an evident influence from the occipital–posterior region towards the channels located in the frontal area of the scalp. Similarly, the significant connections obtained from the window of 4 s (Figure 6b) show similar connectivity patterns exhibiting a greater involvement of the sources located in the posterior part of the scalp, being directed towards the frontal area as well.

In the case of the eyes-open condition for the window of 20 s, the distribution of the channels is different, and by observing its connectivity diagram (Figure 7a), only a few channels show strong connections given the DTF amplitudes, which leads to the idea that the connectivity, in this case, is more uniform among the clusters and tends to have more midrange connectivity amplitudes than the eyes-closed case.

## 4. Discussion

In this study, we explored the stationary characteristics of EEG signals of resting-state conditions through the iterative segmentation of multivariate time series. It was intended to be an intermediate step in the effective connectivity estimation applied to brain activity. Considering the fourth-order central moment known as kurtosis allowed us to quantify how different the EEG sampling distributions were for a density formed by the samples of a pure stationary time series (i.e., Gaussian distribution). This was performed at different levels of segmentation, and such comparison permitted us to assess the effect of the short-time stationarity characteristics affected by this procedure. In this way, the stationary features did not change drastically over time, ensuring uniform characteristics along with the signal. From this, a searching strategy was designed that was dependent on the number of channels from the EEG recordings and the relative variance of the kurtosis distributions, from which the selection of a common window duration across the signals, subjects, and conditions was performed to then assess the effective connectivity.

As has been highlighted throughout this paper, according to the theoretical considerations from some studies [16,34,44,45], the use of an appropriate segment duration to perform the MVAR model-fitting process for EC is an issue that needs to be considered to guarantee consistent results over different experimental setups and inter-subject analysis. Therefore, this research topic is encouraged since the connectivity results are heavily affected by this parameter. In this context, our approach provides a way to make an informed decision regarding the window duration that could be employed in this regard.

As explained throughout the methods, our approach relied on the piecewise subdivision of the time series. This process was individually performed on each signal composing the EEG recording, which according to the mathematical generalization we presented, also allowed the scalability of multiple signals from which segments´ durations were categorized using a matrix representation. From such a generalization, it was possible to estimate different statistical quantities (see Equation (1)), opening the possibility to describe the data with other metrics or extract features from the short-time sequences that resulted from the segmentation.

As briefly mentioned in this paper, despite the importance of the first- and second-order statistics, these are not useful to characterize the segments. Considering that the signals were high-pass filtered in a previous stage using a cutoff frequency of 0.5 Hz, the first-order moment (i.e., the mean) of a segment was reduced to zero, making its value not useful for the characterization of a process. Moreover, the second-order central moment (i.e., the variance) under the same conditions is equivalent to the mean squared, which does not provide any insights into the dispersion of the samples composing the segment. Similarly, the coefficient variation ($\sigma_{x}/\mu_{x}$) is very sensitive to the changes in the first-order statistics, making it grow abruptly as the mean tends to zero. Thereby, only high-order moments such as skewness and kurtosis contribute to the process characterization. Nevertheless, since the normalized version of the kurtosis accounts for the existing difference with a normal distribution when compared to the PDF that is formed from the samples that belong to a segment, this fourth-order moment can be employed to determine the non-stationarity behavior exhibited by a segment of fixed duration [37].

The representation adopted in our approach based on the kurtosis variance serves as a dimensionality-reduction procedure where a vector composed of $t/w_{l}$ kurtosis values calculated from a single EEG signal is re-expressed by the data dispersion. By considering this, similar variance values calculated from the multivariate time series at different segment durations could be compared, and a way to do it is by examining the distribution formed by all the kurtosis variances. From such a PDF, a searching algorithm was designed and the segments with similar variance were grouped and organized so that we could examine how many signals shared the dispersion set by the searching interval limits. Since similar variances from different signals and window durations comprise closer expected values in the kurtosis domain, as shown in Figure 2b, it is expected that the deviations in the kurtosis variance domain to be small for the narrower searching intervals, as shown for the 20%, 30%, and 40% trends in Figure 3a. In such conditions, the searching interval limits are considered stiff and as a consequence, the number of signals meeting the searching parameters is not large (e.g., see the 20% where there are scarce dots). In this sense, by setting up a threshold on the minimum number of channels needed per searching interval, we can settle the trade-off and let the algorithm find the variance interval where most of the channels exhibit similar characteristics and, after grouping and ordering according to the window durations, we can find the shorter duration that captures the most common characteristics across channels.

Since our approach relies on the data that are being explored, it could be considered a non-parametric method for estimating an appropriate window duration to perform the MVAR fitting process. In this way, it is possible to consider conditions related to different types of brain activity and not limited to resting-state analysis in “non-epoched” datasets. This is an advantage in the processing scheme; however, it would need to be optimized to be presented as a third-party tool, e.g., a plug-in for EEGLAB.

The representation of the number of channels as a function of the searching interval and the window duration, shown in Figure 5, provides a useful way to perform a visual inspection of the results obtained from a dataset. It provides insights in a graphical way into what segment duration gathers more channels when a searching interval is as narrow as possible. By setting the threshold on the minimum number of channels, the window duration is found and according to the assumptions discussed so far as it ensures similar stationary properties according to the kurtosis values. In this sense, Table 2 complements the information and gives the exact number of channels from the total available in the dataset following the stationary conditions.

Table 2 gathers as a reference the change in the number of channels when fixed variance percentages are evaluated at every 10% increase of the total. These 10% steps allow us to briefly analyze the variation in the number of channels, but this being a nonlinear process, a smoother variation of such a number would be achieved when percentage steps are narrowed and placed between 50% and 60%, for example, so the exact number of channels in between is achieved.

By considering large steps in the variance, the window durations for each of the subjects are shown in Table 3. The statistical mode of the results suggests that a four-second window for the resting-state conditions under analysis is enough to guarantee similar stationary characteristics across subjects. This result is a useful resource for the EC analysis framework since the window size impacts the connectivity analysis as described in [16,44,45]. Moreover, the window size selection is not usually addressed in the state of the art, which is the reason why many research works use different durations to perform EC as evidenced by the references named throughout this paper.

The characterization from the connectivity allowed us to find common observations with some other works that are closely related to the methodologies implemented, as explained in the work performed by Olejarczyk et al. [22]. In this case, by applying their approach to our data and performing the connectivity analysis, the window of 20 s highlighted central nodes located in the posterior, left central–frontal joint, and prefrontal areas, which were not present in their results. Moreover, the statistical analysis allowed us to find a significant increment of the strength parameter over the alpha band by comparing the conditions of eyes closed and eyes open. Such an increase was mainly produced in some of the regions of higher centrality (prefrontal and central areas).

Our results, differently than in other research works [22,23,24], associated broader scalp areas differentiated by the EEG channels in resting-state conditions with open and closed eyes. In particular, the results point to the participation of more nodes located in the central–parietal and parietal–occipital regions considering the channel network for the alpha frequency band (see Figure 6 and Figure 7). This finding is reflected by the number of connections (i.e., the cost) among the considered channels, suggesting that areas covered by these nodes are possibly involved in facilitating the communication flow between the posterior area and the frontal region. Such speculation could explain the involvement of intermediate structures in the frontal–central joint towards the central–posterior one, thus establishing the importance of the posterior region’s influences on the frontal zone.

These observations provide a starting point in the attempt to characterize the awareness state during relaxation, thus without attention or concentration, by considering the EC measurements related to the alpha frequency band. In fact, the alpha rhythms (8–13 Hz) play an important role in resting states (in either open- or closed-eyes conditions), possibly clearing sensory information from distractors, as well as in waiting states before performing attentional or cognitive tasks.

As explained previously, effective connectivity is more noticeable in the posterior areas as evidenced by the increased network cost for the closed-eyes condition compared with the open-eyes case. These are characteristics expected from the alpha frequency components obtained from acquisition settings like ours. Of note, analyzing active thinking or the engagement of cognitive tasks (more noticeable over the beta frequency band (13–40 Hz)), despite being important, is out of the scope of this work, mainly due to the nature of the EEG signals at our disposal, and that our application example was defined to have a glance at the EC results that could be obtained after applying our methodology, from which we obtained consistent results.

In summary, our study was aimed at providing a novel method for the selection of a segment duration that improves the effective connectivity framework by introducing the analysis of stationary characteristics from the EEG signals as an intermediate step of the EC approach. Another advantage of our methodology is that it is not restricted to being used only for signals that measure resting states. Therefore, even though adjustments would be required to optimize the algorithms and provide flexibility in employing different sampling rates and channel sizes to reduce processing times, we have devised a methodological framework that is potentially applicable to any EEG configuration and possibly beyond resting-state conditions. Future studies could be further devised to demonstrate the capabilities of the method in characterizing connectivity in subjects with specific pathologies.

Moreover, following what is described in [22,23,24], the hypothesis arises of the involvement of the central–posterior and central–frontal regions as the characteristic areas of the resting-state conditions. According to these works, the Default Mode Network (DMN) that comprises those regions is involved in the synchronization over the alpha band. This hypothesis is reinforced by the results obtained in this work as shown in the last section; however, considering only 12 recordings to confirm the hypothesis or not can be misleading, and the EC analysis in our case is limited by the insufficient data available. Despite this limitation and given the consistency of our results, and the resting-state conditions under analysis, our method would provide a feasible approach for the analysis of pathological conditions highlighting statistically significant relationships among brain signal sources that could potentially complement the analysis of clinical conditions.

Under the framework offered by our methodology, when the process is repeated for different EEG recordings acquired from distinct subjects, our approach allows the integration of the data to perform an inter-subject analysis by examining individual results and selecting the window duration that provides common stationary characteristics, firstly for most of the channels on each recording and secondly across subjects selecting the mode, as shown in Table 3. This could be implemented differently, too; similarly to the methods applied in group ICA [46,47], where the brain-activity data from different subjects is concatenated forming a single large block of EEG, MEG, or fMRI time series to find independent components, we could apply this step to analyze the common stationarity of a large multivariate set of signals and obtain a single-window duration from this process. Such a procedure is proposed as a future feature of our approach where we will also analyze the differences in the results with the current method.

Moreover, even though our approach considers a basis window of 1 s for the iterative segmentation, it does not contemplate non-integer values for intermediate segments (e.g., *wl* = 1.5 s, 2.5 s, 3.5 s, …) that could improve the results by evaluating stationarity according to the statistical characteristics of the signals. In this way, as future research work, we plan to evaluate the influence of such kinds of segments in our processing scheme, incorporating a larger dataset as well, and improve the characterization of the resting-state conditions through effective connectivity analysis.

Moreover, as explained in [48,49], other metrics such as the Kullback–Leibler (KL) divergence could be employed to estimate the statistical distance between the sample distributions of our segments and a Gaussian PDF. However, pure stationarity exhibited by random time series (where the expected value is zero) in a real case scenario like ours is difficult to achieve, and such statistical distance under these circumstances would constrain our approach, requiring us to find segment durations that exactly follow a normal distribution. In this way, minimizing the distances and setting a threshold that controls the “stationarity level” from the distance difference derived from the window duration would provide a feasible approach to tackle the same problem. This idea is suggested to be implemented as a future work of our research.

Finally, another idea to be implemented is to investigate the signals at source levels. By employing methods such as the low-resolution electromagnetic tomography (LORETA) taking advantage of the multiple EEG recordings, it is proposed to investigate the connectivity relationships directly estimated from underlying brain-activity generators, which could improve our analysis. Being that this work is a starting point of our research, we focused on the methodology to select an appropriate window duration for EC from the EEG signals. This can be enriched by using approaches to solve the inverse problem, providing an electrical source imaging analysis applied to EC.

## 5. Conclusions

The analysis of stationary characteristics of short-time segments of EEG signals is a topic that, despite its importance in effective connectivity analysis, does not receive enough attention to improve the connectivity results. The use of statistical metrics such as kurtosis to quantify the stationarity of a segment, and by introducing a mathematical description for processing multivariate processes coming from high-density electroencephalography recordings, it contributes to the assessment of the variation of statistical characteristics over time from the different signals. Moreover, including information from different subjects and conditions allows us to make an informed decision of a common segment length that serves to analyze EC in an inter-subject way, guaranteeing uniform conditions among the EEG datasets and conditions.

In addition, the results of our application example showed that uniform characteristics maintained over time with a given segment length provide comparable results to other research works in the literature and other insights that are worth investigating by considering more data, which unfortunately for this case were insufficient to confirm or not the involvement of specific regions in the brain regarding the EC analysis. In this sense, analyzing more data in the analysis is needed to improve the results, in addition to including EEG recordings of other brain activities that could enrich the assessment of the methodology presented here.

## Figures and Tables

**Table 1:** Recordings’ characteristics before and after preprocessing (Discarded recordings are highlighted in gray).

	R1—Opened Eyes					R2—Closed Eyes				
Subject	Raw Signals Duration [s]	Clean Signals Duration [s]	Clean Signals Percentage	Selected Channels	Channel Selection Percentage	Raw Signals Duration [s]	Clean Signals Duration [s]	Clean Signals Percentage	Selected Channels	Channel Selection Percentage
S1	305	46	15%	51	82%	311	172	55%	53	85%
S2	308	235	76%	59	95%	314	256	82%	60	97%
S3	284	46	16%	59	95%	204	43	21%	58	94%
S4	192	101	53%	54	87%	278	123	44%	53	85%
S5	308	205	67%	57	92%	306	155	51%	59	95%
S6	308	230	75%	57	92%	311	226	73%	60	97%
S7	353	135	38%	54	87%	313	212	68%	55	89%
S8	315	197	63%	61	98%	319	231	72%	62	100%
S9	189	145	77%	55	89%	184	116	63%	58	94%
S10	303	172	57%	60	97%	328	240	73%	61	98%
Mean	286	151.2		57		287	177.4		58	

**Table 2:** Segment duration and the number of channels sharing kurtosis variance features for subject S2 in the resting states. The numbers in red correspond to the channels sharing similar stationary values.

Variance Percentage	10%		20%		30%		40%		50%		60%		70%		80%		90%		100%	
Resting State	R1	R2	R1	R2	R1	R2	R1	R2	R1	R2	R1	R2	R1	R2	R1	R2	R1	R2	R1	R2
Low lim.	0.15	0.17	0.142	0.168	0.132	0.165	0.113	0.163	0.086	0.131	0.028	0.065	−0.02	0.025	−0.057	−0.01	−0.107	−0.05	−0.16	−0.83
Upp. lim	0.15	0.172	0.158	0.174	0.168	0.177	0.187	0.179	0.214	0.211	0.272	0.277	0.32	0.317	0.357	0.354	0.407	0.389	0.46	1.172
1 s	0	0	0	0	0	0	0	0	0	0	4	6	20	19	37	32	52	46	59	60
2 s	2	0	1	3	5	8	12	12	28	39	47	60	57	60	58	60	59	60	59	60
3 s	3	2	8	4	12	10	24	11	34	44	42	59	45	60	53	60	57	60	59	60
4 s	3	1	5	2	11	5	20	6	29	26	37	57	43	60	47	60	55	60	59	60
5 s	1	1	2	4	8	7	15	9	20	33	51	59	56	60	56	60	56	60	59	60
6 s	1	2	6	4	12	5	22	9	35	48	45	60	50	60	56	60	57	60	59	60
7 s	1	1	5	3	8	4	16	6	30	40	44	60	48	60	50	60	57	60	59	60
8 s	4	2	4	3	6	6	8	9	25	43	50	60	55	60	57	60	59	60	59	60
9 s	1	0	2	1	4	1	12	2	22	13	55	49	58	60	59	60	59	60	59	60
10 s	2	2	4	3	7	7	13	8	28	25	57	60	59	60	59	60	59	60	59	60

**Table 3:** Selected segment lengths for each subject and resting-state condition.

Subject	Condition	Lower Lim.	Upper Lim.	Variance %	Win Length	Remaining channels	Channel %
S2	R1	0.028	0.272	60%	5 s	47	78%
	R2	0.131	0.211	50%	2 s	39	65%
S5	R1	0	0.156	30%	5 s	41	72%
	R2	0	0.197	40%	4 s	29	49%
S6	R1	0	0.138	30%	4 s	35	61%
	R2	0	0.134	40%	4 s	48	80%
S8	R1	0	0.098	30%	4 s	33	54%
	R2	0.032	0.102	30%	4 s	27	44%
S9	R1	0	0.241	50%	3 s	50	91%
	R2	0.135	0.273	67%	2 s	48	83%
S10	R1	0	0.088	30%	4 s	45	75%
	R2	0.006	0.086	30%	4 s	47	77%
			Mean	41%		Mean	69%

## Data Availability

Not applicable.
